# *Chlamydia* Serine Protease Inhibitor, targeting HtrA, as a New Treatment for Koala *Chlamydia* infection

**DOI:** 10.1038/srep31466

**Published:** 2016-08-17

**Authors:** Amba Lawrence, Tamieka Fraser, Amber Gillett, Joel D. A. Tyndall, Peter Timms, Adam Polkinghorne, Wilhelmina M. Huston

**Affiliations:** 1 Institute of Health and Biomedical Innovation, Queensland University of Technology, Kelvin Grove, QLD, 4059, Australia; 2 Centre for Animal Health Innovation, Faculty of Science, Health, Education and Engineering, University of the Sunshine Coast, Maroochydoore, QLD, 4558, Australia; 3 Australia Zoo Wildlife Hospital, Beerwah, QLD, 4519, Australia; 4 New Zealand’s National School of Pharmacy, University of Otago, Dunedin 9054 New Zealand; 5 School of Life Sciences, University of Technology Sydney, Broadway, NSW, 2007, Australia

## Abstract

The koala, an iconic marsupial native to Australia, is a threatened species in many parts of the country. One major factor in the decline is disease caused by infection with *Chlamydia*. Current therapeutic strategies to treat chlamydiosis in the koala are limited. This study examines the effectiveness of an inhibitor, JO146, which targets the HtrA serine protease for treatment of *C. pecorum* and *C. pneumoniae in vitro* and *ex vivo* with the aim of developing a novel therapeutic for koala *Chlamydia* infections. Clinical isolates from koalas were examined for their susceptibility to JO146. *In vitro* studies demonstrated that treatment with JO146 during the mid-replicative phase of *C. pecorum* or *C. pneumoniae* infections resulted in a significant loss of infectious progeny. *Ex vivo* primary koala tissue cultures were used to demonstrate the efficacy of JO146 and the non-toxic nature of this compound on peripheral blood mononuclear cells and primary cell lines established from koala tissues collected at necropsy. Our results suggest that inhibition of the serine protease HtrA could be a novel treatment strategy for chlamydiosis in koalas.

Despite the growing recognition of their plight, population numbers of Australia’s iconic native marsupial species, the koala (*Phascolarctos cinereus*), continue to decline in large parts of eastern Australia. Population numbers are in decline due to loss of habitat^1^, motor vehicle trauma^2^ and domestic dog attacks^3^. Infectious diseases also place a serious burden on this marsupial. The most clinically significant cause of infectious disease in the koala is the obligate intracellular bacterial parasite, *Chlamydia* (Reviewed in Polkinghorne *et al.*^4^). Two species are known to infect koalas, *Chlamydia pecorum* and *Chlamydia pneumoniae*, with the former being the more pathogenic^5^. *C. pecorum* presents as ocular disease, including discharge, conjunctival and corneal inflammation^6^; or urogenital tract disease, including cystitis, urinary incontinence (termed colloquially as ‘wet bottom’) and fibrosis which can cause infertility^7^. However, the koala *C. pecorum* infection outcome does not always appear to result in pathology as population studies have also shown high chlamydial infection loads in the absence of significant overt disease^8^,^9^. The clinical manifestations of *C. pneumoniae* include a range of severe respiratory signs including difficulty in breathing, sneezing and coughing, and purulent nasal discharge^10^.

Koalas are specialized *Eucalyptus* herbivores who exhibit unique physiological, reproductive and dietary characteristics, including the ability to ingest and metabolize toxic plant metabolites such as phenolic compounds and terpenes^11^. Their unusual metabolism results in limited efficacy of antibiotic treatment, as their efficient hepatic metabolism has been proposed to increase the rate of elimination of some therapeutic drugs^12^. Antibiotic treatments commonly used in humans (such as the tetracycline and macrolide antibiotics) cause emaciation in koalas and typically result in ineffective clearance of chlamydial infections of the lower genital tract^13^,^14^. Currently the most effective and commonly used, systemically administered, chlamydial antibiotic therapy for koalas with urinary-genital tract infections^7^,^15^ is Chloramphenicol 150, (Ceva Delvet, Seven Hill, Australia). This antibiotic has been observed clinically have little detrimental effects on the koala’s specialized gut microflora compared to other medications. When administered at a dose rate of 60 mg/kg once each day for 45 days, this product results in cessation of shedding of *Chlamydia* within two weeks of treatment^13^. However, production of this product is limited, and recent shortages and sporadic manufacturing have resulted in the inability to treat some animals presenting to animal hospitals with chlamydial infections, with many requiring euthanasia as a consequence of disease progression (Personal Communication, Claude Lacasse, Veterinary Services Manager, Australian Wildlife Hospital).

All chlamydiae share a developmental cycle that consists of transitions between two main cell types, the infectious non-dividing extracellular form, known as the elementary body (EB), and an intracellular replicative form, called the reticulate body (RB)^16^,^17^. EBs are responsible for dissemination of infection by attaching to and invading susceptible cells. Upon infection, EBs are internalized in membrane bound vacuoles termed inclusions. EBs differentiate into RBs, the metabolically active form that repeatedly replicate before differentiating back to the infectious EB form. The host cell then lyses, releasing EBs that infect neighbouring cells. If this developmental cycle is disrupted under stressful growth conditions, such as immunological responses, antibiotics or nutrient deprivation, a long term growth that consists of aberrant RB’s that neither replicate, nor differentiate into EBs can result in a process termed persistence (reviewed in Abdelrahman & Belland^17^).

High temperature requirement A (HtrA), is a serine protease, which has been demonstrated to be critical for virulence and intracellular survival of many bacteria^18^,^19^,^20^. We recently demonstrated that *C. trachomatis* HtrA (CtHtrA) is essential for the replicative phase of this organism^21^. Utilising a screening strategy, we identified a serine protease inhibitor (JO146) that binds to HtrA and inhibits the protease activity^21^. The addition of JO146 to the mid-replicative phase of the *C. trachomatis* developmental cycle was lethal *in vitro* and no obvious toxicity was detected in mouse or human cells. The compound was also found to be effective against *C. trachomatis* in a broad range of host cell types^21^. Similar results for JO146 were also obtained for several other species in the genus *Chlamydia*^21^. In this study, we provide the basis for a novel therapeutic strategy for the treatment of chlamydiosis in koalas using the HtrA protease inhibitor, JO146. Our results suggest that this could be an effective target and that further optimization of JO146 could provide valuable new drugs for use in treating the koala.

## Results

### Characterization of the *in vitro* growth characteristics of koala *C. pecorum* urogenital and ocular isolates

**Figure 1:**
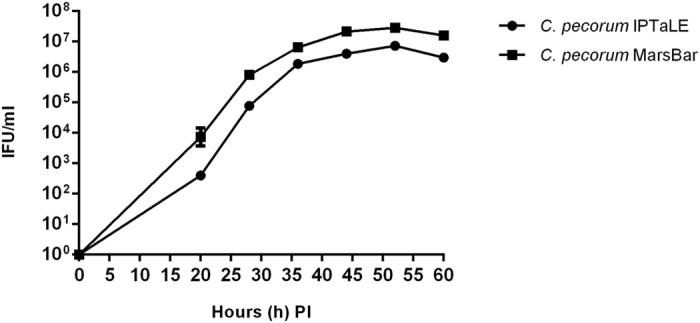
*C. pecorum* IPTaLE and *C. pecorum* MarsBar one step growth curve. *C. pecorum* IPTaLE and *C. pecorum* MarsBar were harvested at 20, 28, 36, 44, 52 and 60 h PI (as indicated on the x-axis) to measure inclusion forming units. Error bars indicate the standard error of the mean obtained from triplicate infected wells (MOI 0.3).

Our previous work suggested that JO146 was most efficacious when added during the mid-replicative growth phase of *C. trachomatis*^22^. However, there are no published growth curves for koala *Chlamydia* isolates so we first needed to profile the growth of these isolates in order to subsequently evaluate inhibitor activity. Growth curves were conducted for urogenital isolates *C. pecorum* MarsBar and *C. pecorum* DBDeUG, and ocular isolate *C. pecorum* IPTaLE^23^. Formation of infectious units were measured at 20, 28, 36, 44, 52 and 60 h PI for *C. pecorum* MarsBar and *C. pecorum* IPTaLE (Fig. 1), and a selection of these time points were used for *C. pecorum* DBDeUG for confirmation of growth similarities for urogenital strains (Supplementary Fig. S1). All isolates demonstrated characteristic bi-phasic chlamydial growth patterns with infectious elementary body (EB) production not detected until 20 h PI and these reached a peak at 36 h PI (Fig. 1). Approximately one-log difference was observed between *C. pecorum* MarsBar and *C. pecorum* IPTaLE EB yield, when measured at 52 h PI (2.85** × **10^7^ IFU/ml and 7.32** × **10^6^ IFU/ml, respectively). The two urogenital isolates *C. pecorum* IPTaLE and *C. pecorum* DBDeUG were consistent in terms of EB yield at 52 h PI (7.32** × **10^6^ IFU/ml and 3.46** × **10^6^ IFU/ml, respectively) (*C. pecorum* DBDeUG data shown in Supplementary Fig. S1).

**Figure 2:**
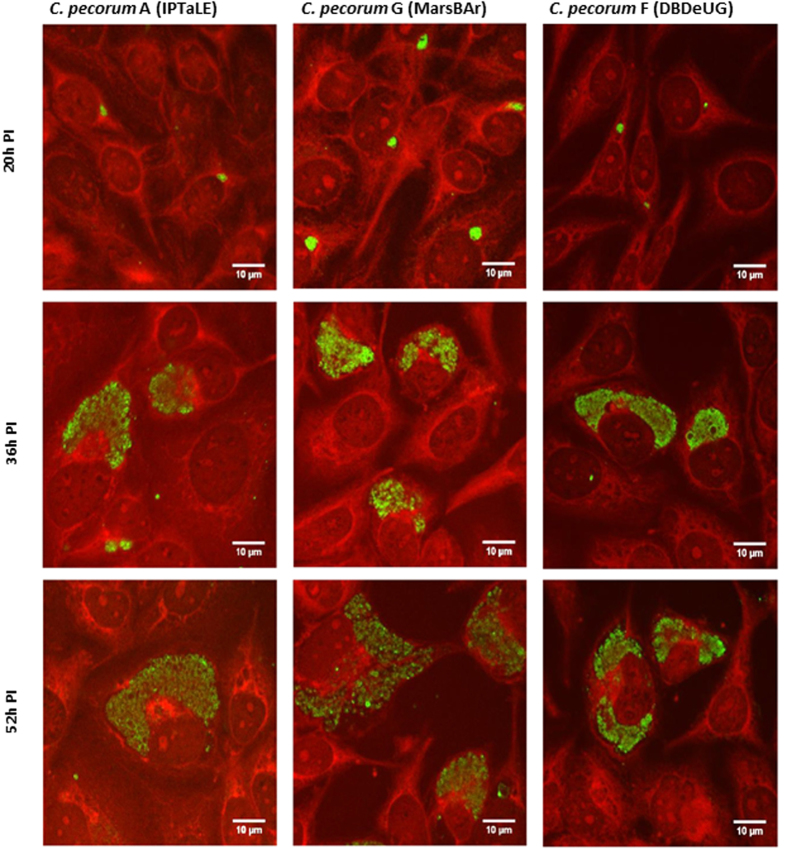
*C. pecorum* inclusion vacuole size shows similar morphology among isolates from different anatomical sites. Cultures were infected at an MOI 0.3. Cultures were fixed and labelled at times indicated to the left of the figure. *Chlamydia* are shown in green and host cells red. Isolates examined were *C. pecorum* IPTaLE, *C. pecorum* MarsBar and *C. pecorum* DBDeUG.

The *Chlamydia* occupy a unique intracellular vacuole termed the inclusion vacuole. The morphology of this vacuole was analysed by confocal microscopy on immuno-labelled cultures at selected time points during the growth curve. Both urogenital strains showed similar inclusion formation and shape (Fig. 2). Initially, inclusions were small and round, located next to the nucleus and increased substantially in size by 36 h PI. Multiple inclusions within a single cell were observed for both strains (Fig. 2). Inclusions were observed to occupy substantial cellular space around the nucleus at 36 h PI onwards. A variety of inclusion morphologies were observed, most often crescent shaped, around the host cell nucleus. Similarly the ocular isolate *C. pecorum* IPTaLE formed small round inclusions at 20 h PI with an increase in inclusion size observed between 20 and 36 h PI (Fig. 2).

### *In vitro* susceptibility testing of koala *C. pecorum* and *C. pneumoniae* isolates to chloramphenicol and tetracycline

We measured the *in vitro* susceptibility of koala *C. pecorum* and *C. pneumoniae* isolates to chloramphenicol and tetracycline. The MICs of tetracycline and chloramphenicol were comparable for both koala *C. pecorum* and *C. pneumoniae* isolates, with all isolates being susceptible to ≤0.25 μg/ml tetracycline and ≤1.00 μg/ml chloramphenicol (Table 1). Both antibiotics were chlamydicidal for all isolates at their determined MIC values were all within one two-fold dilution across all isolates tested (Table 1).

### JO146 addition during the replicative phase of the *Chlamydia* developmental cycle results in significant loss of infectious progeny

**Figure 3:**
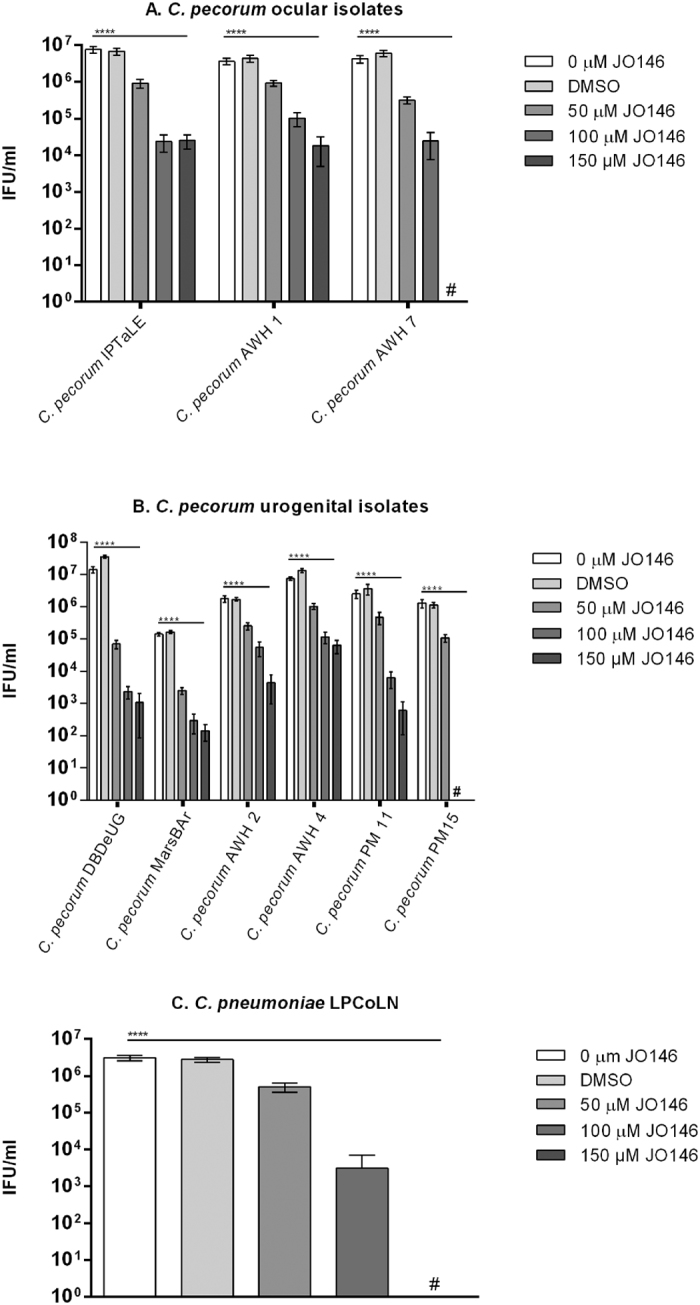
JO146 inhibition during mid-replicative phase of the *Chlamydia* developmental cycle. Infectious yield at 44 h PI (*C. pecorum*) and 72 h PI (*C. pneumoniae*) of isolates treated with JO146 at mid-replicative phase (MOI 0.3). Error bars indicate the standard error of the mean obtained from experimental replicates (minimum of 3). The different koala *Chlamydia* isolates tested were **(A)** *C. pecorum* ocular isolates; *C. pecorum* IPTaLE, *C. pecorum* AWH1, and *C. pecorum* AWH7, **(B)** *C. pecorum* UGT isolates; *C. pecorum* DBDeUG, *C. pecorum* MarsBar, *C. pecorum* AWH2, *C. pecorum* AWH4, *C. pecorum* PM11, and *C. pecorum* PM15, and **(C)** *C. pneumoniae* The concentration of JO146 used is indicated by grey scale shading (legend to the right). The presence of # indicates when the treatment was lethal and no inclusion forming units were detected.

As described previously, JO146 is effective in the mid-replicative phase of the *Chlamydia* developmental cycle^21^. Mid-replicative phase for the *C. pecorum* isolates was demonstrated to be 16 h PI (see Fig. 1). This was estimated based on the observed peak in EB production at approximately 36 h. Mid-replicative phase for the *C. pneumoniae* LPCoLN was determined from data previously published^24^. Infectious progeny (or inclusion forming units; IFU/ml) was determined by passaging the cultures at completion of the developmental cycle after treatment. As shown in Fig. 3, JO146 treatment resulted in complete or highly significant (p** **< 0.0001) loss (up to 10^3–4^ log) of progeny (IFU) when added during the replicative phase. In addition, there was a considerable amount of variability in susceptibility of the isolates at higher doses of JO146. For example, *C. pecorum* MarsBar had 10^2^ IFU/ml at 150 μm JO146, while a similarly urogenital clinical isolate *C. pecorum* AWH4 had a 10^5^ IFU/ml at 150 μm. The activity was most effective at higher doses, with 150 μM being lethal to *C. pecorum* AWH7 (Fig. 3A), *C. pecorum* PM15 (Fig. 3B), and *C. pneumoniae* LPCoLN (Fig. 3C).

### JO146 treatment is most effective when maintained in the culture throughout the replicative and transition to infectious phase of the developmental cycle

In order to understand the chlamydial developmental cycle factors that impact on treatment efficacy, we conducted further *in vitro* experiments varying the length of JO146 treatment. Firstly, we tested short exposures limited to the peak replicative phase, and secondly, extended exposures to ensure the compound is not simply slowing the developmental cycle. Previously, treatments of *C. trachomatis* D/UW-3/Cx showed JO146 to be most effective when maintained in the culture throughout the replicative and transition to infectious developmental cycle phase (elementary bodies)^21^.

**Figure 4:**
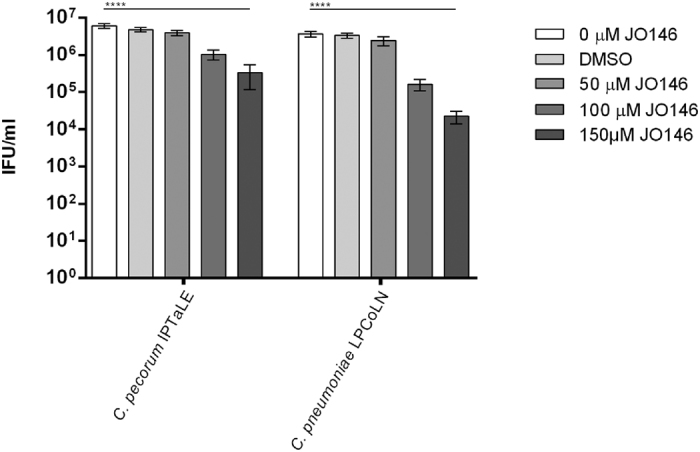
Infectious progeny yield at completion of the chlamydial developmental cycle following 8 h treatment with JO146. Media, JO146 (50, 100, 150 μM) and DMSO was added to *C. pecorum* IPTaLE and to *C. pneumoniae* LPCoLN cultured at an MOI of 0.3 in McCoy B and HEp-2 cells respectively, at 16 h PI and left for 8 h. All wells were then washed with pre-warmed media and further incubated. The cultures were harvested for subsequent measurement of infectious progeny at the completion of the developmental cycle (*C. pecorum* IPTaLE 44 h PI, *C. pneumoniae* LPCoLN 72 h PI). Error bars indicate the standard error of the mean obtained from triplicate infected wells.

We added JO146 to cultures of *C. pecorum* IPTaLE and *C. pneumoniae* LPCoLN at 16 h PI and subsequently removed it from the culture by extensive washing at 24 h PI. We determined Chlamydial viability at 44 h PI (*C. pecorum* IPTaLE) and 72 h PI (*C. pneumoniae* LPCoLN; Fig. 4) and found that JO146 was only slightly effective on *C. pecorum* IPTaLE when washed out 8 h after treatment (24 h PI) with only a 1-log reduction in viability. The compound had a greater effect on *C. pneumoniae* LPCoLN when washed out 8 h after treatment (24 h PI), with a 2-log reduction in viability. The ability of these isolates to recover after treatment with JO146 for 8 h demonstrates a need for the compound to be maintained in culture throughout the replicative phase and into the infectious phase of the developmental cycle.

**Figure 5:**
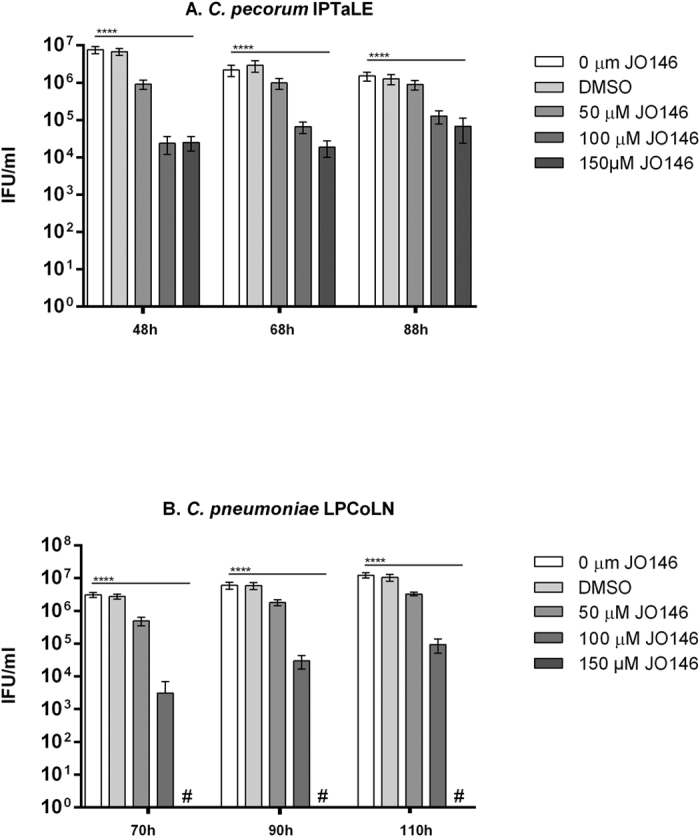
Infectious yield after treatment with JO146 for extended time. Media, JO146 (50, 100, 150 μM) and DMSO was added to **(A)** *C. pecorum* IPTaLE and to **(B)** *C. pneumoniae* LPCoLN cultured at an MOI of 0.3 in McCoy B and HEp-2 cells respectively, at 16 h PI. The cultures were harvested for subsequent measurement of infectious progeny at time points extending further than completion of their developmental cycle. Shown are mean IFU/ml with error bars indicating the standard error of the mean obtained from triplicate infected wells.

To examine the long term stability of the drug, we determined if infectivity can be rescued by extended culturing after exposure to the drug. We added JO146 to cultures of *C. pecorum* IPTaLE and *C. pneumoniae* LPCoLN at 16 h PI and measured infectious progeny at 48, 68, and 88 h PI (*C. pecorum* IPTaLE) and 70, 90 and 110 h PI (*C. pneumoniae* LPCoLN; Fig. 5). JO146 treatment at 16 h PI was still effective for *C. pecorum* IPTaLE after extended culturing and was not notably different between the 48–88 h PI exposures. JO146 treatment at 16 h PI for *C. pneumoniae* LPCoLN however, was lethal for chlamydial infectivity, even after extended culturing.

### JO146 treatment blocks chlamydial inclusion vacuole expansion

**Figure 6:**
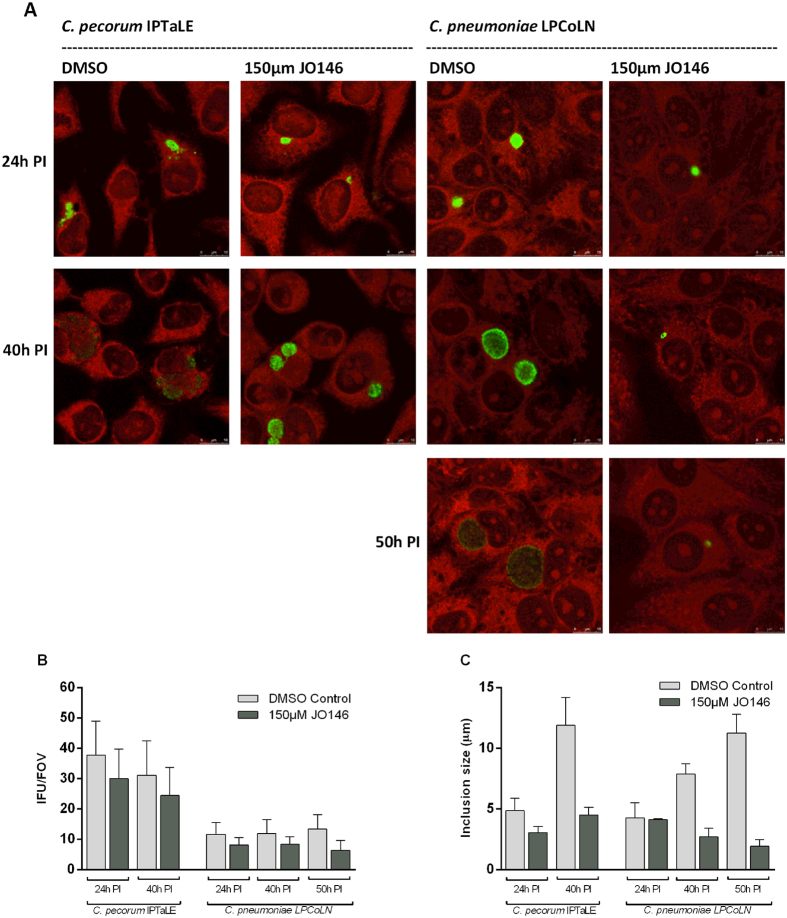
Chlamydial inclusion vacuole size fails to progress over time in cell culture after treatment with 150 μM JO146. **(A)** Cultures treated with 150 μM JO146 or DMSO control at 16 h PI (MOI 0.3). Cultures were fixed and labelled at times indicated to the left and treatment conditions indicated are at the top. *C. pecorum* IPTaLE were cultured in McCoy B cells, *C. pneumoniae* LPCoLN in HEp-2 cells. *Chlamydia* are shown in green and host cells red. **(B)** Quantitative representation of the inclusion size of JO146 and DMSO control treatments at each time point. IFU/FOV = Average Inclusion Forming Units per Field of View ± SE (n = 18). **(C)** Average inclusion size** **± SE where *C. pecorum IPTaLE* (n = 5), *C. pneumoniae* LPCoLN (n = 4).

Using confocal microscopy, we monitored inclusion vacuole size and inclusion numbers after 150 μM JO146 was added to McCoy B grown *C. pecorum* IPTaLE and HEp-2 grown *C. pneumoniae* LPCoLN cultures at 16 h PI. As shown in Fig. 6, *C. pecorum* IPTaLE and *C. pneumoniae* LPCoLN treated cultures did not increase in size compared to control (DMSO) cultures, and at 40 h PI, *C. pecorum* IPTaLE inclusions treated with 150 μm JO146 were 62% smaller than the control while *C. pneumoniae* LPCoLN inclusions treated with 150 μm JO146 at 50 h PI were 83% smaller than control (Fig. 6). In addition, the number of inclusions in the JO146 treated cultures was fewer than that of control for both isolates, suggesting treatment induces loss of inclusions from the cultures (Fig. 6).

In addition, we examined *C. pecorum* cultures by western blot for the HtrA protein compared to β-actin controls. Neither the JO146 treatment or DMSO control treatments had any impact on β-actin levels. However, there was less HtrA protein in the JO146 treatment compared to the DMSO controls 12 (28 h PI) and 26 hours (40 h PI) after the 16 h PI addition of JO146 (see Supplementary Fig. S2).

### JO146 is not cytotoxic for koala peripheral blood mononuclear cells

Previous studies by our laboratory demonstrated that JO146 had no detectable toxicity as measured by cell lysis or metabolic turnover in mouse or human cell lines^21^. To ensure this was also the case for koala cells, we treated fresh koala peripheral blood mononuclear cells (PBMCs) with 100 μM JO146 or DMSO control and assayed cytotoxicity. Cell lysis was assessed using the lactate dehydrogenase assay and metabolic turnover was monitored using the MTS incorporation assay. JO146 was not cytotoxic to koala PBMCs as evident by the lack of cell lysis compared to the control. Metabolic turnover was moderately reduced by approximately 20% for PBMCs treated with 100 μM JO146 (Table 2).

### JO146 is not cytotoxic for koala endometrial and conjunctival cells when cultured *ex vivo*

To determine if JO146 could be a potential therapeutic treatment for koala chlamydiosis, it is essential that the cytotoxicity of the compound be examined directly on koala epithelial cells. As no stable koala cell line currently exists for use in *in vitro* studies, we established primary cell lines for use in this study. We isolated koala primary endometrial and conjunctival cells from epithelium of the koala endometrium and conjunctiva, respectively. We assayed these primary epithelial cells for cytotoxicity after treatment with 100 μM JO146 and DMSO controls and found that JO146 was not cytotoxic to koala primary endometrial cells as evident by the lack of cell lysis compared to control. Metabolic turnover was reduced by approximately 23% for koala primary endometrial cells treated with 100 μM JO146 while only 10% reduction in metabolic activity was detected for the DMSO treated control (Table 2). Similarly for the koala primary conjunctival epithelial cells we observed no toxicity (no cell lysis), but metabolic turnover was reduced by approximately 4% (100 μM JO146 and DMSO control was reduced by 1%) (Table 2).

### JO146 significantly reduced infection of koala primary epithelial cell lines with *C. pecorum* isolates

**Figure 7:**
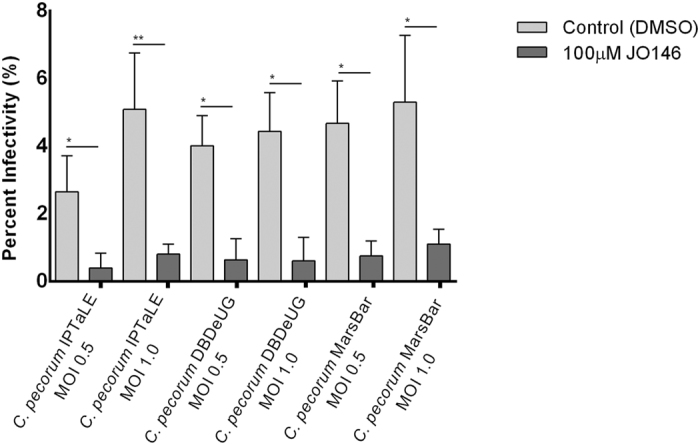
JO146 efficacy on *Chlamydia* infected koala endometrial primary epithelial cells. Uterine primary epithelial cells were extracted from the uterus of a euthanized koala and infected with *C. pecorum* IPTaLE, *C. pecorum* DBDeUG, or *C. pecorum* MarsBar at an MOI of 0.5 or 1.0 and treated at 16 h PI with 100 μM JO146 or DMSO control. Shown is the mean percent infectivity (at 30 h PI, or 14 h after JO146 addition) with error bars indicating the standard error of the mean obtained from triplicate infected wells. The x axis indicates the different koala *Chlamydia* isolates and MOI.

As a final test of the potential efficacy of JO146 in a relevant cellular infection we treated koala primary endometrial cells infected with *C. pecorum* IPTaLE, *C. pecorum* DBDeUG or *C. pecorum* MarsBar at an MOI (multiplicity of infection) of 0.5 or 1.0 with 100 μM JO146 or DMSO control at 16 h PI. As readout of the infection we measured the percentage of cells infected with chlamydial inclusions 35 h PI. JO146 treatment resulted in a significant (p** **< 0.1) reduction in the percentage of cells with chlamydial inclusions at both MOIs tested and for all three isolates in primary *ex vivo* koala endometrial cell cultures (Fig. 7).

## Discussion

This study presents the first stage of development of a new therapeutic for koala chlamydiosis. With an increasingly limited availability of the front-line antibiotic commonly used to treat koala chlamydiosis, a new treatment is urgently needed. We have demonstrated significant reduction (and in some cases, lethality) in infectious progeny of *Chlamydia* from koala clinical cases of disease using both in *in vivo* and *ex vivo* cell cultures.

*In vitro* susceptibility patterns of antibiotics across chlamydial *spp*. and hosts appear uniform^25^,^26^,^27^, and indeed, our results for susceptibility of koala *C. pecorum* isolates appear consistent with activity at or below the range previously reported (1–2 μg/ml)^28^. These results indicate that the plasma concentrations of chloramphenicol reached in the koala (reaching a peak plasma concentration of 3.02 μg/ml on day 1 of treatment, and 4.82 μg/ml by day 15^12^) are sufficient for treatment. However, for koalas affected by severe chlamydial disease, antibiotics alone are not sufficient to cure the clinical symptoms^12^. In addition, production of the commonly used antibiotic chloramphenicol is limited. Although there is a potential commercial alternative treatment (Florfenicol), pharmacokinetics, efficacy or safety studies for this drug in koala chlamydiosis has not been carried out^28^. Therefore, alternative treatment strategies are required that not only treat the disease, but also limit side effects given the unique biology of the koala.

Very little *in vitro* growth data is available for koala *C. pecorum* or any strains of this species. Prior to assessing the efficacy of our therapeutic agent, it was necessary to analyse the growth characteristics of ocular and urogenital koala *C. pecorum* strains. Growth curves have previously demonstrated that human *C. trachomatis* ocular and genital strains have quite distinct kinetics, with the ocular strains generally considered to be slow growing strains^29^. We tested three koala *C. pecorum* strains from ocular and urogenital sites and found that there were differences in the observed growth kinetics. Specifically, the *C. pecorum* IPTaLE (ocular) isolate has a longer lag phase and lower overall yield of infectious progeny (EBs) compared to the others. Genetically, the three strains are >98% identical with variances limited to two regions; the plasticity zone and an area encoding polymorphic membrane proteins, where SNPs were found to cause premature stop codons in six *C. pecorum* genes^23^. Expanded *in vitro* and genetic studies of additional clinical isolates will be required to establish whether genital and ocular koala *C. pecorum* strains share similar tropisms to their human *C. trachomatis* counterparts.

As a first step to evaluating the efficacy of our HtrA protease inhibitor JO146, we treated cultures during the replicative phase (estimated to be ~16 h PI) of the koala *C. pecorum* and *C. pneumoniae* developmental cycle. This treatment resulted in significant loss of infectious progeny for many koala *C. pecorum* isolates and lethality for two koala *C. pecorum* isolates and koala *C. pneumoniae*. Analysis of the chlamydial cell morphology during treatment supported this loss of infectious progeny, with inclusion size failing to progress (and in some cases diminishing) within the treated cultures. As previously shown by our laboratory, mid-replicative phase addition of JO146 is detrimental to many species of *Chlamydia* including *C. trachomatis* D, *C. trachomatis* L2, *C. pecorum* IPA, *C. suis* and *C. caviae* and in many host cell types^21^,^30^. Here we show that JO146 treatment of *C. pecorum* IPTaLE and *C. pneumoniae* LPCoLN was most effective when maintained in the culture throughout the replicative and transition to infectious phase of the developmental cycle. This indicates that lead drug candidates will need to be optimized for tissue stability and bio-availability with overall extended half-life *in vivo*.

The removal of JO146 at 24 h PI (8 h after treatment) showed only a 1-log (*C. pecorum* IPTaLE) and 2.5-log (*C. pneumoniae* LPCoLN) reduction in infectious progeny. However, when the compound was maintained in the media throughout the replicative phase, a 3-log (*C. pecorum* IPTaLE) and complete loss (*C. pneumoniae* LPCoLN) of infectious progeny resulted. Importantly, with extended culture of *C. pecorum* IPTaLE (68 and 88 h PI), there was no rescue of infectious progeny and *C. pneumoniae* LPCoLN was similarly not able to recover at 90 and 110 h PI. This result suggests a sustained effect of JO146 treatment and further supports its activity as being primarily bactericidal. This result could also suggest that the targets of JO146 (HtrA most likely but possible other serine proteases) are required for not only the replicative phase but also for the differentiation from reticulate bodies to the infectious elementary body form of *Chlamydia*.

Our previous research using JO146 supported that HtrA is essential for *Chlamydia trachomatis* serovar D during the replicative phase of the developmental cycle^22^. These previous data suggested that *Chlamydia* HtrA functions in critical protein maintenance and assembly; possibly for vital outer membrane virulence factors, based on some of our biochemical data^31^,^32^. This means that for *Chlamydia* at least HtrA is an essential virulence factor in addition to a well-known protein stress response function in many bacteria. However, this is not necessarily unexpected^33^, as HtrA is also an essential virulence factor for *Legionella*^33^.

A key finding of our study was the ability of JO146 to kill *Chlamydia* in koala primary *ex vivo*-cell cultures. There are no published reports of culturing Koala clinical isolates of *Chlamydia* in primary *ex vivo* koala cells and this method represents an important step forward in the context of an urgent need for new antibiotics. JO146 significantly reduced the number of infected cells in cultures of *C. pecorum* isolates in koala primary endometrial cells. Unfortunately, the primary cultures were not able to be maintained and amplified sufficiently to passage the *Chlamydia* and measure the infectious progeny on repeat infections. Due to limits in the availability of freshly necropsied koala tissue, additional replicates of this experiment could also not be performed. Nevertheless, the results of this single *ex vivo* treatment of *C. pecorum* infections with JO146 are consistent with those obtained *in vitro*.

Importantly, cytotoxicity assays performed on freshly harvested koala PBMCs, endometrial and conjunctival cells confirmed overall low toxicity of JO146. We did observe a measurable reduction in proliferation induced by JO146 in all *ex vivo* primary cell cultures. However, it is hard to evaluate the implication of this result, given that the cells were not able to be maintained beyond three passages in all experiments attempted. This reduction may not occur in more stable primary *ex vivo* cells, which may or may not be relevant *in vivo*, hence further studies are required to assess the implications of this result.

Overall, our studies of JO146 have thus far shown that it can significantly reduce the infectious progeny of clinical isolates of koala chlamydial. The compound is lethal to many clinical isolates and induced several log reductions in the remaining isolates. The compound is non-cytotoxic to koala PBMCs, conjunctival, and endometrial primary *ex vivo* tissue, maintains stability in culture extending beyond the *Chlamydia* infectious cycle, and remained bactericidal throughout treatment. Although these results are promising, this compound in its current formulation, is not completely lethal to all isolates, therefore continued optimization will be required. Further studies are currently underway to generate more potent derivatives of JO146.

This is the first report of a protease inhibitor successfully being applied *in vitro* and *ex vivo* to treat koala *C. pecorum* and *C. pneumoniae*, and validates JO146 or other inhibitors derived from JO146 as possible options for treating koala chlamydiosis. Here we have provided the basis for future studies investigating the efficacy of JO146 and further derivatives *in vivo*.

## Methods

### Ethics Statement

Koala *Chlamydia pecorum* strains isolated in this study were collected from koalas presenting to Australia Zoo Wildlife Hospital and Port Macquarie Koala Hospital as a part of their routine veterinary care and diagnosis. The use of these samples for *Chlamydia* culturing was considered by the University of the Sunshine Coast Animal Ethics Committee (USCAEC) and determined to be exempt from requiring further approval (AN/E/14/01). Koala tissues for primary epithelial cell culture were collected from two koalas, presenting to Australia Zoo Wildlife Hospital with injuries or disease. The animals were euthanized as a result of their condition and necropsy subsequently performed. The use of these koala tissues for this purpose was considered and also exempted from requiring further approval (AN/E/15/06).

### Isolates used in this study

The ovine *C. pecorum* polyarthritis strain IPA (ATCC VR629), originally isolated from the joint fluid of a sheep in Iowa, USA^34^, *C. trachomatis* D (D/UW-3/Cx) and *C. trachomatis* L2 (L2-434/Bu), both obtained from the ATCC, were used as controls in this study. The isolates in Table 3 were all collected from captive and/or wild koalas located in Queensland and New South Wales, Australia. Swab samples were collected and stored in SPG transport media at point of care and frozen at −80 °C. The koala *C. pecorum* isolates were propagated in McCoy B cells and passaged no more than five times. All isolates have been confirmed by 16S RNA PCR and sequencing to be positive for *C. pecorum*^8^.

### *Chlamydia* culture

*C. pecorum* and *C. trachomatis* isolates were routinely cultured in McCoy B cells on DMEM, 10% Fetal calf serum (FCS), 0.1 mg/ml streptomycin, and 0.05 mg/ml gentamycin. *C. pneumoniae* was routinely cultured in HEp-2 cells on DMEM, 10% FCS, 0.1 mg/ml streptomycin, and 0.05 mg/ml gentamycin. Inhibitor experiments were routinely conducted in 96-well plates seeded with 20,000 host cells per well 24 h prior to chlamydial infection. All strains were cultured in 37 °C incubators with 5% CO_2_ and, unless otherwise stated, all infections were routinely conducted at a Multiplicity of Infection (MOI) of 0.3. The Inclusion Forming Units (IFU) was determined from cultures harvested at the completion of the developmental cycle during which inhibitor treatment was conducted (time of harvest is indicated on the figure). Briefly, JO146 and DMSO control (JO146 solvent), at doses indicated on the graphs (always at a 1 in 1000 dilution factor), was added at 16 h post infection (PI) and left in the cultures until completion of the developmental cycle (or at time points indicated). Harvested cultures were lysed by vigorous pipetting and serially diluted onto fresh monolayers and fixed and stained at 30 h PI for enumeration of IFU/ml^21^. The compound JO146 was commercially sourced as previously described^30^.

### Antimicrobial Susceptibility Testing of Koala *Chlamydia* isolates

Antimicrobial susceptibility testing of koala *C. pecorum* and *C. pneumoniae* isolates in McCoy B and HEp-2 cell lines respectively, was performed as previously described^35^ with chloramphenicol and tetracycline on 96-well microtiter plates without passage (MIC) and by one passage (MCC). The inoculum size of infectious *Chlamydia* for all MIC and MCC comparisons was 10,000 IFU/well. The MIC was defined as the concentration of antibiotics one two-fold dilution more concentrated than the MIC_TP_ (where MIC_TP_ is defined as the concentration of antibiotics where 90% of inclusions or greater displayed altered size or morphology). The MCC is defined as the lowest concentration of drug that produces no morphologically normal inclusions by one freeze-thaw passage in 96-well microtiter plates.

### Microscopy

*C. pecorum* and *C. pneumoniae* cultures were examined using immunofluorescence with a Leica SP5 Confocal microscope with a 63**×** oil objective. Coverslip cultures were washed with PBS and fixed with 100% methanol for 10 min. Cultures were stained with fluorescein isothiocyanate (FITC)-labelled *Chlamydia*-specific anti-lipopolysaccharide (anti-LPS) monoclonal antibody and host cells stained with Evan’s blue (Cell labs, Australia) and then mounted on slides in Prolong Gold (Invitrogen) prior to visualization.

### Isolation of koala Peripheral Blood Mononuclear Cells

Koala Peripheral Blood Mononuclear Cells (PBMC’s) were isolated as previously described^36^. Briefly, the PBMC fraction was isolated by Ficoll-Paque density gradient centrifugation and washed twice with phosphate-buffered saline (PBS). The pellet was then resuspended in RPMI (10% FCS, 0.1 mg/ml streptomycin, and 0.05 mg/ml gentamycin) before seeding into 96-well plates 12 h prior to use in cytotoxicity assays. All wells were stimulated with a final concentration of 100 μg/ml Phorbol Myristate Acetate (PHA) at this time.

### Isolation of koala primary epithelial cells

Tissue from the conjunctiva and endometrium were collected from two koala’s euthanized due to illness at Australia Zoo Wildlife Hospital. Harvested tissue was placed in HBSS media with 0.2% collagenase D and a homogenate prepared. The tissue was washed twice with HBSS and further treated with 2 U/ml DNase, with 10% FCS added to stop the DNase activity. The tissue was then spun and the pellet resuspended in red blood cell lysis buffer before being washed twice with complete keratinocyte-SFM and finally resuspended in complete keratinocyte-SFM, 10% fetal calf serum, 0.1 mg/ml streptomycin, 0.25 mg/ml Fungizone and 0.05 mg/ml gentamycin. Cells were passaged no more than two times before use in cytotoxic assays and JO146 efficacy assays.

### Cytotoxicity and Proliferation Assays

Koala PBMC’s and primary conjunctival and endometrial cells were assayed for cytotoxicity after treatment with JO146 and DMSO controls using the lactate dehydrogenase assay for cell lysis (CytoTox 96^®^ Non-Radioactive Cytotoxicity Assay, Promega). Metabolic turnover was monitored using the MTS incorporation assay (CellTiter 96^®^AQueous Non-Radioactive Cell Proliferation Assay, Promega).

PBMCs were collected from anticoagulated blood from four different koalas. Epithelial cells were extracted from endometrial tissue of two koalas for primary culture. Conjunctival epithelial cells were extracted from conjunctival tissue of one koala for primary culture. DMSO control was used at the same volume of DMSO as that added for 100 μM JO146 treatments. PBMC’s were stimulated with a final concentration of 100 μg/ml PHA at 12 h prior to treatment with JO146.

### Statistical Analysis

All graphing and statistical analysis was conducted using the Prism GraphPad Software. ANOVA with multiple comparisons were conducted relative to the DMSO or cell control for statistical analysis, ****indicates *p*** **< 0.0001 or greater significance was identified.

## Additional Information

**How to cite this article**: Lawrence, A. *et al.* *Chlamydia* Serine Protease Inhibitor, targeting HtrA, as a New Treatment for Koala *Chlamydia* infection. *Sci. Rep.* **6**, 31466; doi: 10.1038/srep31466 (2016).

## Supplementary Material

Supplementary Information

## Figures and Tables

**Table 1:** *In vitro* susceptibility testing of koala *C. pecorum* and *C. pneumoniae* isolates.

Isolate (Koala)δ	MICα (μg/ml) Tetracycline	MCCβ (μg/ml) Tetracycline	MICα (μg/ml) Chloramphenicol	MCCβ (μg/ml) Chloramphenicol	MCCβ (μg/ml) JO146
*C. pecorum* IPTaLE (Ocular)	0.250γ	0.250	0.750	0.500	>90
*C. pecorum* AWH1 (Ocular)	0.125	0.125	0.500	0.500	>90
C. pecorum AWH7 (Ocular)	0.250	0.125	0.500	0.500	60
C. pecorum DBDeUG (Urogenital)	0.250	0.250	0.750	0.750	>90
C. pecorum MarsBar (Urogenital)	0.250	0.250	1.000	0.750	>90
C. pecorum AWH2 (Urogenital)	0.125	0.125	0.500	0.500	>90
C. pecorum AWH4 (Urogenital)	0.250	0.250	1.000	1.000	>90
C. pecorum PM11 (Urogenital)	0.250	0.125	1.000	0.500	>90
C. pecorum PM15 (Urogenital)	0.125	0.125	0.500	0.500	60
*C. pecorum* IPA	0.250	0.250	1.000	1.000	>90^30^
C. pneumoniae LPCoLN	0.250	0.125	1.000	0.750	60
C. trachomatis D/UW-3/Cx	0.500	0.500	1.000	1.000	>90^30^
*C. trachomatis* L2-434/Bu	0.250	0.250	1.000	1.000	>90^30^

^*α*^MIC is defined as the concentration of antibiotic one two-fold dilution more concentrated than the MIC_TP_ (where MIC_TP_ is defined as the concentration of antibiotic where 90% of inclusions or greater displayed altered size or morphology).

^β^MCC is defined as the lowest concentration of antibiotic that produces no morphologically normal inclusions by one freeze-thaw passage in 96-well microtiter plates.

^γ^MIC antibiotic range tested was 0.008 μg/ml–2.000 μg/ml, and cultures were infected with no less than 10,000 IFU/well.

^δ^*C. pecorum* infections were fixed at 30 h PI while *C. pneumoniae* were fixed at 68 h PI.

**Table 2:** Cytotoxicity and proliferation of cells treated with JO146.

**Koala PBMC LDH Assay - % cytotoxicity (n** = **4)**α	
**100 μM JO146**§	**DMSO**
−3.10** **± (3.41)	1.77** **± (3.02)
**Koala PBMC MTS Assay - % proliferation (n** = **4)**α	
**100 μM JO146**	**DMSO**
19.74** **± (3.041)	0.29** **± (1.23)
**Koala Uterine Tissue LDH Assay - % cytotoxicity (n** = **2)**α	
**100 μM JO146**§	**DMSO**§
−3.96** **± (3.56)	−8.70** **± (6.17)
**Koala Uterine Tissue MTS Assay - % proliferation (n** = **2)**α	
**100 μM JO146**	**DMSO**
23.62** **± (0.20)	10.54** **± (1.52)
**Koala Conjunctival Tissue LDH Assay - % cytotoxicity (n** = **1)**α	
**100 μM JO146**§	**DMSO**
−10.76	−18.69
**Koala Conjunctival Tissue MTS Assay - % proliferation (n** = **1)**α	
**100 μM JO146**	**DMSO**
3.86	1.03

^α^Experiments were performed in triplicate and the results expressed as percentage cell death compared with untreated control cells by using the formula [1 − (mean absorbance of treated cells/mean absorbance of untreated cells)] * 100. Results are shown with SEM indicated in parenthesis.

^§^Negative values indicate treated cultures had less cytotoxicity/more proliferation than control.

**Table 3:** Koala *C. pecorum* and *C. pneumoniae* isolates used in this study.

**Animal Code**	**Symptoms of chlamydial disease**	**Anatomical site**	**Geographical location**
*C. pecorum* IPTaLE 23	Conjunctivitis	Eyes	Ipswich, QLD
*C. pecorum* DBDeUG^23^	Cystitis	UGT	Deception bay, QLD
*C. pecorum* MarsBar^23^	Cystitis	UGT	Mt Cotton, QLD
*C. pecorum* AWH 1	Conjunctivitis	Eyes	Lake Somerset, QLD
*C. pecorum* AWH 2		UGT	
*C. pecorum* AWH 4	Cystitis	UGT	Teddington, QLD
*C. pecorum* AWH 7	Conjunctivitis	Eyes	Noosa, QLD
*C. pecorum* PM 11	N/A	UGT	Port Macquarie region, NSW
*C. pecorum* PM15	N/A	UGT	
*C. pneumonia* LPCoLN^10^	Respiratory illness	Nasopharynx	Brisbane, QLD
