# Supplementary material for: Chlamydia Serine Protease Inhibitor, targeting HtrA, as a New Treatment for Koala Chlamydia infection
Source: Sci Rep. 2016 Aug 17;6:31466. doi: 10.1038/srep31466 (PMC4987629; doi:10.1038/srep31466)
Supplement: Supplementary Information [file srep31466-s1.pdf]

SUPPLEMENTARY INFORMATION

"*Chlamydia* Serine Protease Inhibitor, targeting HtrA, as a New Treatment for Koala *Chlamydia* infection"

Authors: Amba Lawrence[1], Tamieka Fraser[2], Amber Gillett[3], Joel Tyndall[4], Peter Timms[2], Adam Polkinghorne[2], and Wilhelmina M. Huston[1,5 *]

Supplementary Figure S1. *C. pecorum* DBDeUG one step growth curve. Shown are mean IFU/ml with error bars indicating the standard error of the mean obtained from triplicate infected wells (MOI 0.3). *C. pecorum* DBDeUG was harvested at 20, 36 and 52 h PI (as indicated on the x-axis).

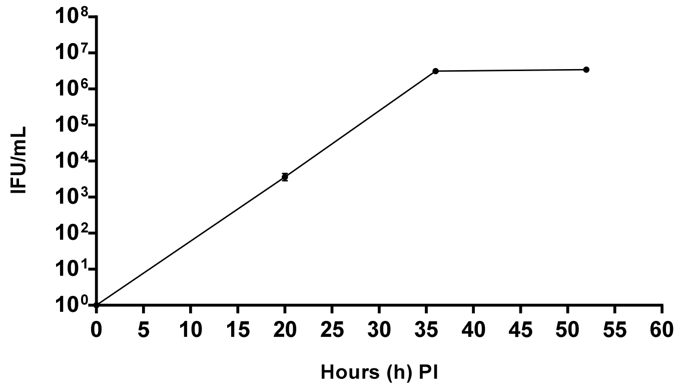

Supplementary figure S2 and Method

HtrA protein levels are reduced after JO146 treatment.

Method: *C. pecorum* cultures were conducted in 6 well culture plates at MOI of 0.5 on McCoyB cells. JO146 treatment and DMSO controls were added at 16 h PI. The cultures were harvested at 12 (28 h PI) and 26 hours (40 h PI) after the 16 h PI addition of JO146 or DMSO. The cultures were harvested in 1x SDS PAGE loading buffer, boiled and western blots conducted for HtrA and β-actin protein.

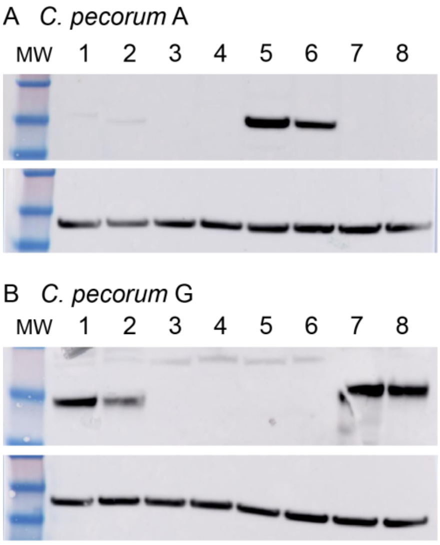

Supplementary Fig S2. HtrA protein levels are reduced after JO146 treatment but not β-actin. The samples are **A.** *C. pecorum* A Molecular weight marker: 1. JO146 24 h PI (infected), 2. DMSO 24 h PI (infected), 3. JO146 24 h PI (uninfected), 4. DMSO treated 24 h PI (uninfected), 5. JO146 40 h PI (infected), 6. DMSO 40 h PI (infected), 7. JO146 40 h PI (uinfected)), 8. DMSO 40 h PI (uninfected). HtrA IB top, β-actin bottom. **B.** *C. pecorum* G. A Molecular weight marker: 1. JO146 24 h PI (infected), 2. DMSO 24 h PI (infected), 3. JO146 24 h PI (uninfected), 4. DMSO treated 24 h PI (uninfected), 5. JO146 40 h PI (uninfected), 6. DMSO 40 h PI (uninfected), 7. JO146 40 h PI (infected)), 8. DMSO 40 h PI (infected). HtrA IB top, β-actin bottom.
